# Cucumber Green Mottle Mosaic Virus Decreases Chlorophyll *a* Content in Cucurbit Crops by Upregulating the Key Gene in Chlorophyll Catabolic Pathway, *Chlorophyllase 1*

**DOI:** 10.3390/plants14193086

**Published:** 2025-10-06

**Authors:** Zhenggang Li, Yafei Tang, Guobing Lan, Lin Yu, Shanwen Ding, Zifu He, Xiaoman She

**Affiliations:** Guangdong Provincial Key Laboratory of High Technology for Plant Protection, Plant Protection Research Institute, Guangdong Academy of Agricultural Sciences, Guangzhou 510640, China; lizhenggang@gdppri.com (Z.L.); yf.tang1314@163.com (Y.T.); languo020@163.com (G.L.); yulin@gdppri.com (L.Y.); dingshanwen@gdppri.com (S.D.)

**Keywords:** cucumber green mottle mosaic virus, *chlorophyllase 1*, upregulation, chlorophyll, cucurbit crop

## Abstract

Cucumber green mottle mosaic virus (CGMMV, *Tobamovirus viridimaculae*) is a tobamovirus that induces leaf green mottling, mosaic patterns, bleaching, fruit sponginess, rotting, and malformation symptoms in various cucurbit crops. The underlying mechanisms by which CGMMV elicits these symptoms have yet to be elucidated. In the present study, we observed that the infection of CGMMV in bottle gourd, but not in *N. benthamiana*, led to the significant upregulation of a key gene involved in chlorophyll degradation, *Chlorophyllase 1* (CLH1). This induction may be closely linked to chlorophyll degradation, particularly that of chlorophyll *a* (Clh *a*) in bottle gourd plants. Phylogenetic analysis showed that the amino acid sequence of BgCLH1 has a closer relationship with those of CLH1 from other cucurbit crops and has a relatively farther relationship with those of the well-studied CLH1 from *Arabidopsis thaliana* and *Citrus sinensis*. Further, confocal microscopy analysis indicated that BgCLH1 may be localized to the cytoplasm instead of the chloroplast. Moreover, silencing of the Bg*CLH1* gene not only reduced viral accumulation but also resulted in an increase in chlorophyll content. Similar results were also observed in watermelon, suggesting that this regulatory mechanism may be conserved across cucurbit crops. Our findings thus reveal a complex and intricate interplay between viral infection and the chlorophyll metabolic pathway.

## 1. Introduction

Plant viruses are a category of minuscule organisms that are obligate parasites within their host creatures. To survive in the host cells, viruses can hijack the host metabolism and gene expression pathways to create a favorable environment for virus replication, movement, and assembly. Plants that have been infected by viruses frequently exhibit a range of disease symptoms, such as yellowing, mosaic, leaf curling, mottling, stunted growth, necrosis, and wilting [1]. Virus-encoded proteins can manipulate various physiological pathways within the host plants, including hormone signal transduction, photosynthetic, cell cycle, and reactive oxygen species (ROS), leading to the emergence of disease symptoms.

In the realm of the plant kingdom, chloroplast is the pivotal organelle that carries out photosynthesis, phytohormone synthesis, and immune response. Emerging studies have shown that chloroplast is the prime target for plant viruses. Viral infections often disrupt the structure and function of chloroplasts by interfering with the expression, localization, and function of chloroplast proteins or enzymes involved in photosynthesis, chlorophyll biosynthesis, and carbon metabolism [2,3]. Viruses can often affect the hormone signal pathway to suppress the immune response of host plants. Previous reports have shown that the C4 protein of tomato yellow leaf curl virus (TYLCV) re-localizes from the plasma membrane to the chloroplasts upon the activation of plant defense and interferes with the chloroplast-dependent anti-viral salicylic acid (SA) biosynthesis [4]. The malformation and dysfunction of chloroplasts will eventually contribute to the disease symptom development in host plants.

Cucumber green mottle mosaic virus (CGMMV) is a tobamovirus which is capable of infecting various cucurbit crops [5]. CGMMV can cause leaf green mottling, mosaic, bleaching, fruit sponginess, rotting, and malformation [5]. However, the underlying mechanism remains unknown. In this study, we found that the infection of CGMMV significantly induces the gene expression of *CLH1* in bottle gourd, leading to the reduced accumulation of chlorophyll, especially chlorophyll *a*. Silencing of *BgCLH1* via CGMMV-based gene silencing reduced the viral accumulation and upregulated the chlorophyll accumulation. These results demonstrate that the disease symptoms caused by CGMMV may be due to the degradation of chlorophyll.

## 2. Results

### 2.1. CGMMV Infection Significantly Induced the Expression of BgCLH1 in Bottle Gourd, but Not in N. benthamiana

**Figure 1:**
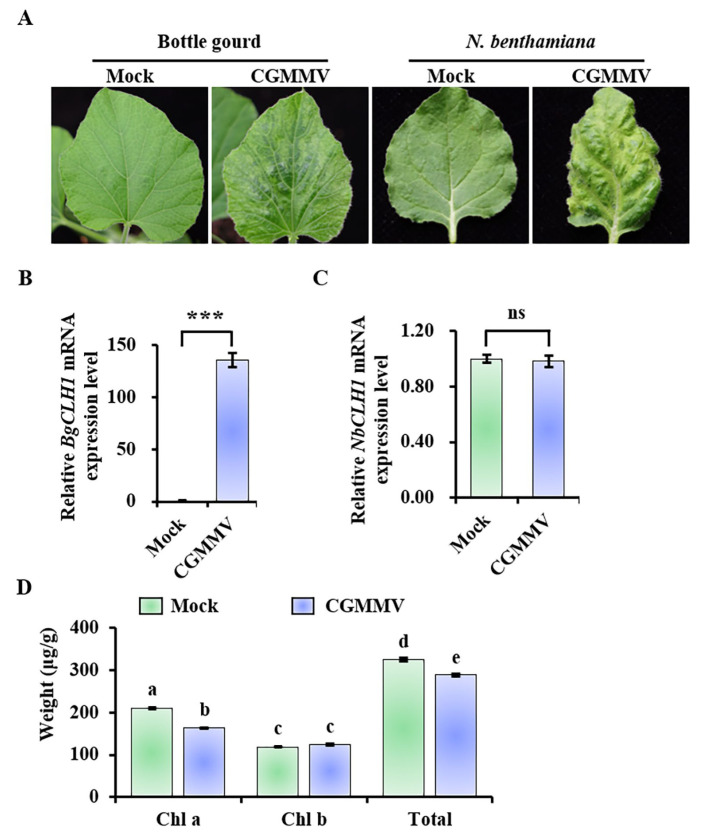
CGMMV induces the expression of *CLH1* and decreases the chlorophyll content in bottle gourd. (**A**) Disease symptoms in the leaves of bottle gourd and *N. benthamiana* infected by CGMMV. Photos were taken at 12 dpi and 10 dpi for bottle gourd and *N. benthamiana*, respectively. The relative mRNA expression level of *CLH1* in bottle gourd (**B**) and *N. benthamiana* plants (**C**). These experiments were repeated three times with at least three plants, respectively, with similar results. Asterisks indicate the *p*-value between the mock and CGMMV-infected leaves according to Student’s *t*-test (two-tailed). *** *p* < 0.001. ns, no significant difference. Error bar indicates the SD. *LsH3* and *NbEF1α* were used as the reference genes in bottle gourd and *N. benthamiana*, respectively. Error bar indicates the SD. (**D**) The content of chlorophyll in bottle gourd leaves. At 12 dpi, the leaves of the mock and CGMMV-infected leaves were taken for chlorophyll measurement by UPLC. Each group contains three repeats. Different letters in the figures indicate significant differences, determined by Duncan’s multiple range test.

CGMMV induces green mottle, yellowing, and mosaic symptoms in bottle gourd and *Nicotiana benthamiana* (Figure 1A). In an effort to unravel the molecular mechanism of these disease symptoms, we conducted transcriptome sequencing on bottle gourd samples at 12 days post inoculation (dpi) with CGMMV [6]. Our analysis revealed a significant upregulation of the bottle gourd *chlorophyllase 1* (*BgCLH1*, Lsi05G009520) at 12 dpi in response to CGMMV infection. Chlorophyllase (Chlase, CLH) is the first and rate-limiting enzyme in the chlorophyll (Chl) degradation pathway. To validate this result, we employed quantitative real-time PCR (qRT-PCR) with primer pair qPCR-BgCLH1-F/R (Supplementary Table S1), which confirmed an over 100-fold increase in *BgCLH1* transcript abundance in the leaves of CGMMV-infected bottle gourd (Figure 1B). In contrast, the expression of the homologous gene in *N. benthamiana*, *NbCLH1*, exhibited no significant alteration (Figure 1C).

Given the established role of CLH1 in the chlorophyll catabolic pathway, we hypothesized a decrease in chlorophyll content in CGMMV-infected bottle gourd plants. To test this, we measured the chlorophyll levels in both infected and mock bottle gourd leaves using ultra-performance liquid chromatography (UPLC). The result showed that the total chlorophyll content, including both Chl *a* and Chl *b,* was reduced by about 12% in CGMMV-infected leaves compared with the mock control (Figure 1D). Notably, this decrease was attributed primarily to a reduction in Chl *a*, with its concentration falling by approximately 20% due to CGMMV infection, while Chl *b* levels remained unaffected (Figure 1D).

### 2.2. Phylogenetic Analysis of BgCLH1

**Figure 2:**
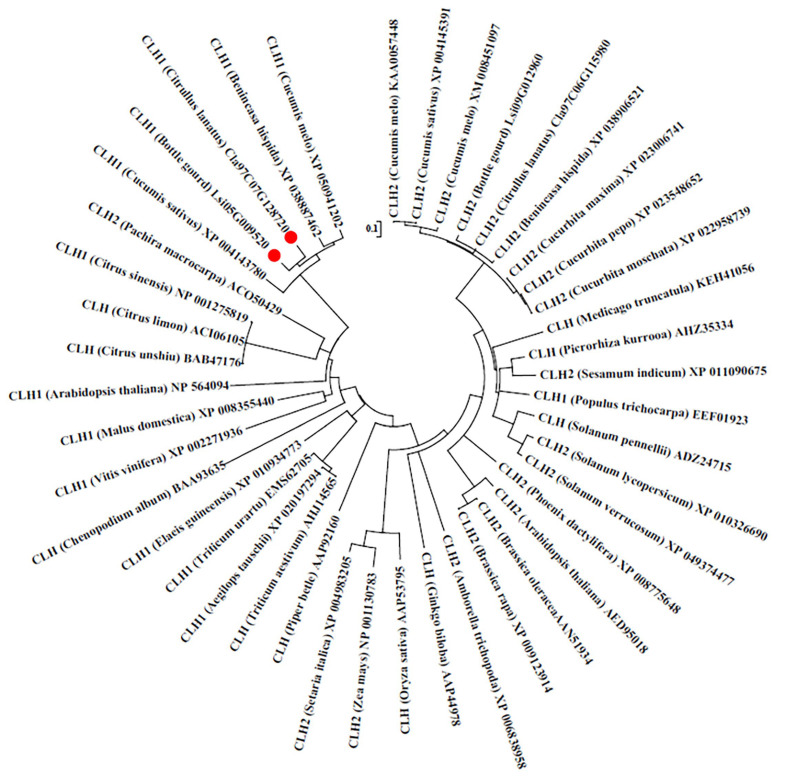
Phylogenetic analysis of CLH1 and CLH2 from different plant species. The amino acid sequences of CLH1 and CLH2 were obtained from the NCBI database. Phylogenetic tree was constructed with MEGA 7 (version 7.0.26) software using the neighbor joining method with 1000 bootstrapped replications. The red dots indicate the CLH1 sequences used in this article.

To analyze the phylogenetic position of BgCLH1 among plant CLHs, the amino acids sequence of BgCLH1 was blasted against NCBI. The results showed that BgCLH1 shares the highest similarity with the CLHs from some cucurbit crops. Further analysis with other amino sequences of CLH1 and CLH2 using the phylogenetic tree also demonstrates that BgCLH1 is close to the CLH1 of *Citrullus lanatus*, *Benincasa hispida*, *Cucumis sativus*, and *Cucumis melo* (Figure 2).

### 2.3. Subcellular Localization of BgCLH1

**Figure 3:**
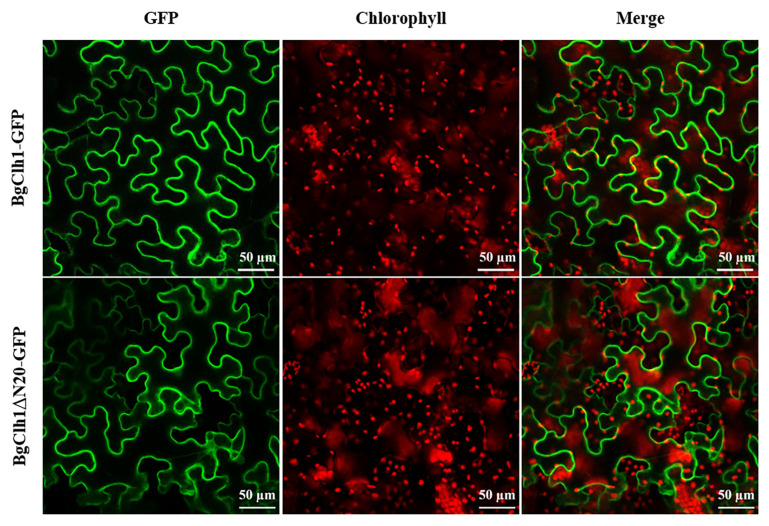
Subcellular localization of BgCLH1 and BgClh1ΔN20. The subcellular localization of BgCLH1 and BgCLHΔN20 was analyzed under a confocal microscope. Bar = 50 μm.

The chlorophyll catabolism pathway is assumed to occur in the chloroplast, where the chlorophyll is localized. Thus, the localization of CLH should also be in the chloroplast. To check the subcellular localization of BgCLH1, GFP fluorescent protein was fused to the C-terminus of BgCLH1. To our surprise, BgCLH1 was localized in the cytoplasm, but not in the chloroplast (Figure 3). Moreover, BgCLH1 with deletion of the first 20 amino acids (BgCLH1ΔN20), which may be the mature version, still presents in the cytoplasm (Figure 3). This result implies that BgCLH1 is localized in the cytoplasm instead of the chloroplast.

### 2.4. Down-Regulation of BgCLH1 Expression Reduced Viral Infection and Increased Chlorophyll Accumulation

**Figure 4:**
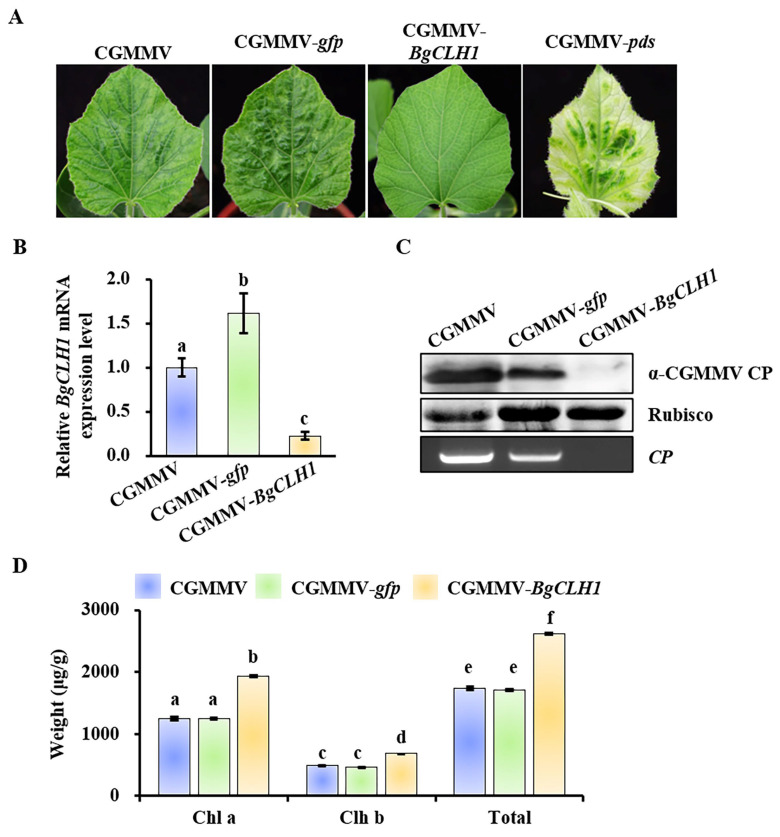
The effect of *CLH1* silencing on the viral accumulation and chlorophyll content in bottle gourd. (**A**) Symptoms of bottle gourd plants infected by CGMMV and other CGMMV derivatives. (**B**) qRT-PCR detection of *BgCLH1* mRNA accumulation in bottle gourd plants from (**A**). This experiment was repeated three times with three plants in each replicate. Different letters indicate the significant differences performed by Duncan’s multiple range test. (**C**) Western blot and RT-PCR detection of CGMMV CP/*CP* accumulation in bottle gourd plants from (**A**). Rubisco was used as equal loading. (**D**) Detection of chlorophyll content in bottle gourd plants from (**A**). Different letters indicate the significant differences performed by Duncan’s multiple range test.

To ascertain whether the reduction in Chl *a* was due to the induction of *BgCLH1*, we employed the CGMMV-based virus-induced gene silencing (VIGS) [7,8] to downregulate the expression level of *BgCLH1*. Bottle gourd possesses two CLH homologs, *CLH1* (Lsi05G009520) and *CLH2* (Lsi09G012960), with a relatively low sequence similarity of just 40% (Supplementary File S1). Then, the fragment of *BgCLH1* was introduced into a CGMMV infectious clone to obtain pCB301-CGMMV-*BgCLH1*. CGMMV, CGMMV-*gfp*, CGMMV-*BgCLH1*, and CGMMV-*pds* were inoculated into bottle gourd plants via agroinfiltration, respectively. At 15 dpi, the upper leaves of bottle gourd plants infected by CGMMV-*pds* exhibited photobleaching, indicating successful gene silencing (Figure 4A). In comparison, plants infected with CGMMV or CGMMV-*gfp* displayed mosaic and yellowing symptoms, with CGMMV-*gfp* showing slightly reduced virulence (Figure 4A). Notably, plants infected with CGMMV-*BgCLH1* showed no significant disease symptoms, suggesting a role of BgCLH1 in symptom development (Figure 4A). qRT-PCR analysis revealed that the expression of *BgCLH1* was downregulated by approximately 80% in plants infected with CGMMV-*BgCLH1* compared to those infected with CGMMV (Figure 4B). Western blot and PCR analyses demonstrate that the viral accumulation of CGMMV was significantly reduced in *BgCLH1*-silenced plants (Figure 4C).

Moreover, to evaluate the effect of *BgCLH1* silencing on chlorophyll content, the upper leaves from these plants were taken for analysis. The results showed that, in contrast with CGMMV and CGMMV-*gfp*, the levels of both Chl *a* and Chl *b* were elevated in *BgCLH1*-silenced plants, with Chl *a* increasing by about 60% and Chl *b* by about 40% (Figure 4D). Collectively, these results underscore the pivotal role of *BgCLH1*, not only in symptom development but also in facilitating CGMMV infection in bottle gourd.

**Figure 5:**
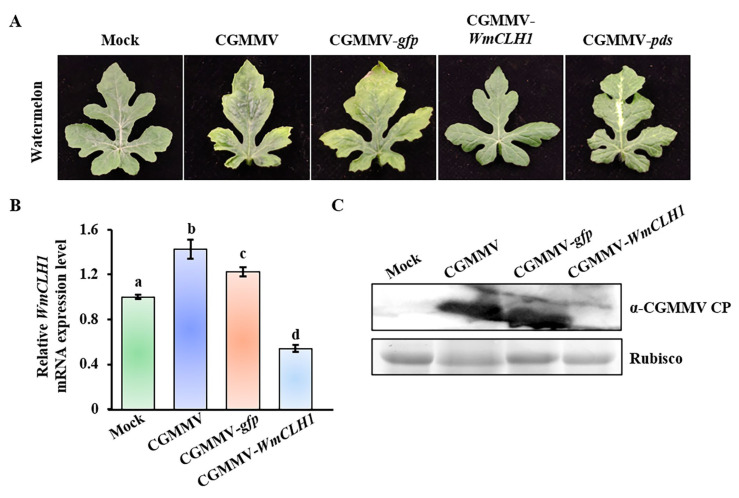
The effect of *CLH1* silencing on the viral accumulation in watermelon. (**A**) Symptoms of watermelon plants infected by CGMMV and other CGMMV derivatives. CGMMV-*pds* was used as positive control. (**B**) qRT-PCR detection of *CLH1* mRNA expression level in plants from (**A**). This experiment was repeated three times, with three plants in each replicate. Different letters indicate significant differences conducted by Duncan’s multiple range test. (**C**) Western blot detection of CGMMV CP accumulation in plants from (**A**).

To further elucidate the role of CLH1 in other host plants of CGMMV, *CLH1* was silenced in another natural host of CGMMV, watermelon. Similarly to bottle gourd, watermelon also encodes two CLH genes, *WmCLH1* (Cla97C07G128720) and *WmCLH2* (Cla97C06G115980), with a sequence identity of approximately 48% (Supplementary File S1). We constructed a CGMMV infectious clone containing a fragment of *WmCLH1*, designated as pCB301-CGMMV-*WmCLH1*. At 15 dpi, watermelon plants infected with CGMMV-*pds* exhibited characteristic white bleaching symptoms, indicative of successful gene silencing (Figure 5A). qRT-PCR analysis revealed that the expression of *WmCLH1* was elevated by about 50% in response to CGMMV infection, whereas in plants infected with CGMMV-*WmCLH1*, the expression was reduced by about 50% (Figure 5B). Furthermore, Western blot analysis demonstrated a significant reduction in viral accumulation in *WmCLH1*-silenced plants (Figure 5C). These findings suggest that the function of CLH1 during CGMMV infection may be a conserved feature among cucurbit crops, but not in *N. benthamiana*.

## 3. Discussion

Previous studies have highlighted a strong correlation between the disease symptoms caused by viruses and the process of chlorophyll biosynthesis. For instance, the SP protein of rice stripe virus (RSV) has been shown to alter the subcellular localization of the PsbP protein from the chloroplast to the cytoplasm, leading to changes in the structure and function of the chloroplasts [9]. Cucumber mosaic virus (CMV) Y satellite RNA produces short interfering RNAs (siRNAs) to downregulate the expression of a key gene in chlorophyll synthesis, magnesium protoporphyrin chelatase subunit I (*ChlI*), leading to the yellowing symptoms in host plants [10]. Tobacco mosaic virus (TMV) *flavum* strain causes strong yellow and green mosaic symptoms by a reduction in specific proteins of the PSII core complexes, but not by a reduction in pigment biosynthesis [11]. In addition, a report revealed that the interaction between TMV replicase protein and *Arabidopsis* Aux/IAA protein PAP1 can interfere with the plant’s auxin response system to induce specific disease symptoms [12].

In this study, we found that CGMMV infection induced the expression of chlorophyllase 1 (CLH1) in bottle gourd, but not in *N. benthamiana*. In virus-infected plants, the accumulation of Chl *a* decreased, but the accumulation of Chl *b* remained the same compared with the mock control. CLH plays an essential role in the degradation of chlorophyll by catalyzing the hydrolysis of phytol from chlorophyll to chlorophyllide [13,14]. CLH was discovered in 1912, but the gene (*CLHs*) that encodes the protein with the CLH activity was not identified until 1999 [15,16,17]. Both Chl *a* and Chl *b* can be the substrates of CLH, but a report showed that CLH has preferential activity towards Chl *a* [18,19]. Previous reports have shown that the infection of cucumber mosaic virus (CMV) increased the chlorophyllase activity, leading to more chlorophyll breakdown in infected plants [20,21]. Furthermore, another study showed that African cassava mosaic virus (ACMV) upregulates the chlorophyll degradation genes, including CLH, pheophytinase (PPH), and pheophorbide a oxygenase (PaO), leading to the reduced accumulation of Chl *b* [22].

We found that BgCLH1 was localized in the cytoplasm, not in the chloroplast. Previous reports have shown that the N-terminal deletion sequence (21 amino acids for *citrus* and 30 amino acids for *Chenopodium*) is the mature protein of CLHs [23]. However, BgCLH1 is still localized to the cytoplasm with deletion of the first 20 amino acids. Prediction using online tools (https://services.healthtech.dtu.dk/services/SignalP-6.0/ (accessed on 5 July 2025) and https://services.healthtech.dtu.dk/services/DeepLoc-2.1/ (accessed on 5 July 2025) shows that BgCLH1 does not contain any characteristic signal peptide, and BgCLH1 is a soluble protein mostly localized in the cytoplasm. Previous studies have demonstrated that although most CLHs are localized to the chloroplast, some CLHs does not contain predicted chloroplast transit peptides and may not be localized in the chloroplast [24]. CLH encoded by *Chenopodium album* contains a typical endoplasmic reticulum (ER) transit peptide instead of a chloroplast transit peptide, suggesting that CaCLH may also be localized in other cell organelles besides the chloroplast [16,23]. Both AtCLH1 and AtCLH2 are not localized in the chloroplast, and later studies showed that they were localized in the ER and tonoplast [25,26]. Moreover, CLH1 protein of *Chlamydomonas reinhardtii* was also found to be localized outside of the chloroplast [27].

Silencing of CLH1 not only increased the chlorophyll accumulation including both Chl *a* and Chl *b* but also significantly reduced the viral accumulation. A previous study showed that expression of citrus ChlaseΔN, the mature protein of chlorophyllase lacking the N-terminal 21 amino acids, results in a dramatic chlorotic phenotype in squash plants under low-intensity light and a lesion-mimic phenotype under natural light [13]. A report demonstrated that AtCLH1 is involved in plant damage control, as silencing of *AtCLH1* increased susceptibility to the necrotrophic fungus *Alternaria brassicicola* [28]. Further results suggested that AtCLH1 is not responsible for chlorophyll breakdown in intact leaf tissue but promotes the chlorophyllide formation upon the disruption of leaf cells, which may be a defense process against chewing herbivores [25]. Since BgCLH1 does not localize in the chloroplast, it is inferred that BgCLH1 may have other functions besides degrading the chlorophyll.

Our findings contribute to this body of knowledge by showing that CGMMV infection triggers the upregulation of the chlorophyll degradation gene, *CLH1*, leading to a reduction in chlorophyll accumulation, particularly Chl *a*. This regulatory mechanism may be conserved among cucurbit crops, which are the natural hosts of CGMMV, but appears to be distinct in *N. benthamiana*. Our results suggest a nuanced interplay between viral infection and the chlorophyll metabolic pathway, with implications for understanding the complex dynamics of plant-virus interactions and potentially guiding the development of novel strategies for managing viral diseases in cucurbit crops.

## 4. Materials and Methods

### 4.1. Plant Growth Conditions

Bottle gourd and *N. benthamiana* plants were grown in a controlled growth chamber with a 13 h light/11 h dark photoperiod at 24 °C.

### 4.2. Virus Inoculation and Detection

*Agrobacterium* containing the infectious cDNA clones of CGMMV and CGMMV derivatives was infiltrated into the cotyledons of bottle gourd plants. For *N. benthamiana*, 5-6-leaf-stage plants were used for inoculation. At 12 or 15 dpi, samples were taken for total RNA extraction using TRIzol reagent (Takara, Dalian, China), followed by reverse transcription using PrimeScript RT Master Mix (TaKaRa, Dalian, China). PCR detection of CGMMV was performed with the primer pair CGMMV-CP-F/R (Supplementary Table S1). For Western blot detection, leaves were taken with the same weight and following the same protocol as described previously [29].

### 4.3. Real-Time PCR Analysis

The total RNA of different samples was extracted with TRIzol reagent (Takara, Dalian, China) and followed by the removal of DNA contamination using RNase-free rDNase I (TransGen Biotech, Beijing, China). Then, the treated RNA was subjected to reverse transcription using PrimeScript RT Master Mix (TaKaRa, Dalian, China). Real-time PCR was performed, with the primer pairs listed in the Supplementary Table S1, on a Bio-Rad CFX96 real-time system using TB Green Premix Ex Taq (TaKaRa, Dalian, China). Expression levels of *CLH1* were normalized to *H3* for bottle gourd and watermelon, and to *EF1α* for *N. benthamiana*. Relative transcript levels were calculated using the 2^ΔΔCt^ method, with mock-treated or CGMMV-infected samples as the reference. Three biological replicates and at least three technical replicates were included for each sample.

### 4.4. Plasmid Construction

To make the CLH1 silencing vector, the fragments of *BgCLH1* and *WmCLH1* were amplified using the primer pairs (Supplementary Table S1) from the corresponding cDNAs. Then, the fragments were cloned into *BamHI*-treated pCB301-CGMMV-BamHI-VIGS infectious clones [8]. For BgCLH1-GFP and BgClh1ΔN20-GFP, the full-length nucleotide sequences of BgCLH1 and BgClh1ΔN20 were amplified using primer pairs BgCLH1-pGDGm-F/R and BgCLH1ΔN20-pGDGm-F/BgCLH1-pGDGm-R, respectively. The fragments were introduced into the *ApaI*-digested pGDGm vector [30,31] using a Seamless Assembly Cloning Kit (TransGen Biotech, Beijing, China).

### 4.5. Chlorophyll Measurement

The chlorophyll measurement was performed as described previously [32]. Each sample of each group was taken with equal weight. The separation and quantification of Chl *a* and Chl *b* were performed using HPLC (Agilent 1200, Santa Clara, CA, USA).

### 4.6. Phylogenetic Tree Construction

The phylogenetic tree was constructed using the amino acid sequences of CLH1 and CLH2 with MEGA7 software [33].

### 4.7. Confocal Microscopy

Confocal microscopy was performed using a ZEISS LSM780 confocal microscope (Carl Zeiss MicroImaging GmbH, Jena, Germany). The excitation wavelengths were 488 nm and 633 nm for GFP and chlorophyll, respectively.

## Data Availability

The original contributions presented in this study are included in the article/Supplementary Materials. Further inquiries can be directed to the corresponding authors.

## Supplementary Materials

The following supporting information can be downloaded at https://www.mdpi.com/article/10.3390/plants14193086/s1, Supplementary Table S1: Primers used in this study; Supplementary File S1: The nucleotide sequences of *BgCLH1*, *BgCLH2*, *WmCLH1*, *WmCLH2*, and the nucleotide sequence alignment analysis.
