# Supplementary material for: Cucumber Green Mottle Mosaic Virus Decreases Chlorophyll a Content in Cucurbit Crops by Upregulating the Key Gene in Chlorophyll Catabolic Pathway, Chlorophyllase 1
Source: Plants (Basel). 2025 Oct 6;14(19):3086. doi: 10.3390/plants14193086 (PMC12526093; doi:10.3390/plants14193086)
Supplement: Supplementary file 1 [file plants-14-03086-s001.zip › Li et al-Supplementanry table.pdf]

Supplementary Table S1 | Primers used in this study

Primer	Primer sequence (5'-3')	Purpose
qPCR-BgCLH1-F	F: TGCTTATCTTCACGCCGACCAA	qPCR detection of BgCLH1 mRNA accumulation
qPCR-BgCLH1-R	R: AAGGTTGAGGAAATCAGAGTAGAAGGA	
qPCR-NbCLH1-F	ATCGGCGTTGATCCTGTTGATGG	qPCR detection of NbCLH1 mRNA accumulation
qPCR-NbCLH1-R	AGATTGAAGGAATGAGGAGTATAGGTGAGA	
BgCLH1-pGDGm-F	CAAGCTTGTCGACGGGCCATGGCGGCAGTAATAGTGGT	Construction of BgCLH1 and BgCLH1ΔN20 plasmid
BgCLH1-pGDGm-R	CTTCTCCTTTACTCATGGGCCCTTTAGAGGAGGATGTTTTGTAAAT	
BgCLH1ΔN20-pGDGm-F	CAAGCTTGTCGACGGGCCATGAGTGAAGTTTTTGAAACAG	
CGMMV-BgCLH1-F	CGGGATCCCACAGCCGAGGTGGCAAAACA	Construction of BgCLH1 silencing vector
CGMMV-BgCLH1-R	CGGGATCCTCATCCCAGGCGGGTTGTCGTT	
CGMMV-WmCLH1-F	CGGGATCCCATAGCCGAGGTGGCAAAACA	Construction of WmCLH1

**CGMMV-WmCLH1-R**	**CGGGATCCTAATCCCAGGCGGGTTGTCGTT**	**silencing vector**
**CGMMV-CP-F**	**ATGGCTTACAATCCGATCACACCT**	**RT-PCR detection of CP gene**
**CGMMV-CP-R**	**CTAAGCTTTCGAGGTGGTAGCCT**	
**WmCLH1-qPCR-F**	**CCATTGCTTATCTTCACACCGACCAA**	**qPCR detection of WmCLH1 mRNA accumulation**
**WmCLH1-qPCR-R**	**GGAAGCGATGAGGCGAAGGAGA**	
