# Supplementary material for: Cucumber Green Mottle Mosaic Virus Decreases Chlorophyll a Content in Cucurbit Crops by Upregulating the Key Gene in Chlorophyll Catabolic Pathway, Chlorophyllase 1
Source: Plants (Basel). 2025 Oct 6;14(19):3086. doi: 10.3390/plants14193086 (PMC12526093; doi:10.3390/plants14193086)
Supplement: Supplementary file 1 [file plants-14-03086-s001.zip › Supplementary File S1.pdf]

BgClh1 （Lsi05G009520）：

ATGGCGGCAGTAATAGTGGTGAAAGAAGAGGGGAAGCCAGTTTCAGCGGCGGTGGATCCTAGTGAAGTT
TTTGAAACAGGGAAATTTGAAGTGAGTACCATCACTGTGAAAACAGGCATATTTTCAGCTTCAAAGCCA
TTGCTTATCTTCACGCCGACCAACCCTGGTTCTTATCCAGTCATCTTGTTCCTTCATGGCTTCTCCTGC
GCTGGATCCTTCTACTCTGATTTCCTCAACCTTATAGCTTCCCATGGCTACATTATTGCTGCCCCACAG
TTGTACGTGATGCCAACCACAAGTGAAATGAACGAAATAAACTCAGCATCAGAAGTAATAACATGGTTG
GCGTCGGGACTTGACCCATTACTCCCAGACAAAGTGAAAGGAGATATTTCAAAGCTAAGTCTAGTGGGG
CACAGCCGAGGTGGCAAAACAGCCTTTTCCTTAGCCTTAGGCTACGGCCGCCCTTCCCTTCCCTTCTCC
GCCGTAATCGGCGTCGACCCAGTAGCCGGAACCAAATACTTCCAGCCGGAGCCCCACATCCTAACCCCA
CTCTCCGAACCCTTCAACATTTCCGTACCCATCACAGTCATTGGAACCGGACTGGGCCCCAAGAGAGCG
AACCCAGTCACATGCCCTTGCGCCCCTGTTGGCTACAACCACGTTGAGTTTTTCCAAAAAAGCAAGCCC
AGTTGCGCCCACTTTGTAGCGGTGAATTACGGCCACATGGACATTCTAAACGACAACCCGCCTGGGATG
ACAGGCTTTTTCACGAACATTGCTTGTAAGAATGGGAAAGGCCCAAGAAAGCTTATGAGGAAGTGCTGC
AGTGGGCTTGTGGTTGCTTCTTTGAAGGCTTATCTTGACAATGATGAATCATTTCTGAATGCTATTTAT
ATTGACCCTTCTATTGCTCCTGTTGAGCTAAATCCTGCTGAGGTTATTTACAAAACATCCTCCTCTAAA
TAA

BgClh2 （Lsi09G012960）：

ATGGGGGATGAAAAATTGAAATTGAAACTCCAACAAACCTCACTCGCCACCGCCACCGCCATGGCTATG
GCTACTGCTTCTTTTCCTCCGCCGTCGGCCACCGCCGGCTCTGCAATTTCGTCAAATGTCTTCGAGATC
GGAAAATTCAGCGCGGTGCTTGAAAAGTTCGAACCGGCTGGATGTTGCTCATCTGGCCGATTTCTTCCG
GCTCCCCCGCCGAAGCCGCTGCTGATCGGCCGGCCTTCCGAAGCCGGAGAGTTCCCGGTGCTGCTGCTC
ATCCATGGCTATCTCCTTTACAACACTTTCTACTCTCAGCTCATCCATCACATCGCCTCTCATGGCTTC
ATCTTGTACTCTGTGGCCGGACCAGATACAAGCGAAGAAATCAAAGCAACAGCCGCCATAATAAACTGG
CTGCCGAAAGGCCTCCGGCACCTCCTTCCACCGCACGTGAACCCAAACCTAACCAAAATAGCCCTAGCC
GGCCACAGCCGCGGCGGCAAAACTTCCTTCGCCTTAGCCCAACAAAAATCCCCAAAATTGTCAGCCGTA
ATCGGGCTCGATCCCGTCGACGGAACAGGGTCCGGCAAGCAAACCCATCCCCCAGTTCTGAAATACATC
CCCCACTCGTTGGATCTAGGGCTCCCGGTTCTGGTAATCGGGTCGAGTCTGGGAGAATTGAAAAGAAAT
CCACTGTTTCCCCCTTGCGCCCCAAAAGGAATCAACCACGAGGAGTTCTTTAAGGAGTGCCGGAATCCG
GCGTACTATTTTGTTGTGAAGGATTACGGGCATTTGGATTTGTTGGATGATGAAACTGGAGGGATTAGA
GGGAAGGTGAGTTACTGTTTGTGTAAAAATGGGGAATCTAGAGAGCCGATGAGGAGGTTTGTTGGTGGG
GCTGTGGTTGCGTTTTTGAAGGCTTATTTTGATGGGGAAGAAGGAGATTTGAGAGCCATTGAAGATGGG
GATTTGAGCTTGCCTGTTCAACTTCAAACTGTTGAATCTTTTCTCTGA

WmClh1 （Cla97C07G128720）：

ATGGCGGCAATAGTGGTGAAAGAAGCTAAGTCAGTTTTCGAAACAGGAAAATTTGAAGTAACTGCCATC
ACTGTAAAATCAGACATATTTTCAAGTTCAAAGCCATTGCTTATCTTCACACCGACCAACCCAGGTTCG
TATCCCCTCATCTTGTTCCTTCATGGCTTCTCCTGCGCCGGATCCTTCTACTCCCATCTCCTTCGCCCC
ATCGCTTCCCATGGCTACGTTATTGCTGCCCCACAGTTGTACATGATGCCAACCACAAGTGAAATGGAA
GAAATAAAGTCAGCATCAGAAGTGATAACATGGTTGCGGTCGGGACTTAACCCATTACTCCCAAACGAT
GTAAAAGGAGATATTTCAAAGCTAAGTCTAGTGGGCCATAGCCGAGGTGGCAAAACAGCCTTTTCCTTA
GCCTTAGGCTACGGCCGCCCTTCCCTTCCCTTCTCCGCGGTCATCGGCATCGACCCAGTCGCCGGAACC
AAGTGTTTCCAGCCCCACCCCCACATCCTAACCCCACTCTCCGAACCCTTCAACATTTCCGCACCCATC
ACAGTCATTGGAACCGGGCTGGGTCCCGAGAAGGCGAACCTAGTCACATGCCCTTGCGCCCCCGATGGC
TACAACCACGCTGCGTTTTTCCAAAAGAGCAAGCCCACTCGCGCTCACTTTGTGGCGGTGGACTACGGG
CACATGGACGTTCTGAACGACAACCCGCCTGGGATTACGGGCCTTTTCACCAACATTGCTTGTAAGAAC
GGGAAGGGTCCAAGAGAGCTTATGAGGAAGTGCTGCAGTGGACTCGTGGTTGCTTCCTTGAAGGCTTAT
CTTGACAATGATCAATCAATTCTGAATGCCATTTATGTTGACCCTTCCATTGCTCCCGTGGACCTAAAT
CCTGTGGAGCTTATTTACAAACCATCCACTGCACAGGGGCTGATCTATTAA

WmClh2 （Cla97C06G115980）：

ATGGCTATGGCTACTGCTTCTTTTCCTCCGCCGTCGGCCACCGCGCGCTCTGCAGTTTCGTCCAATGTC
TTCGAGATCGGAAAATTCAACGCGGTGCTTGAAAAGGTCGAATCGGCTGGATGTTGCTCATCTGGCCGA
TTTCTTCCGGCTCCTCCGCCGAAGCCGCTGCTGATCGGCCGGCCTTCCGATGCCGGAGAGTTCCCGGTG
CTGCTGCTCATCCACGGCTATCTCCTTTACAACACTTTCTACTCTCAGCTTATCCATCACATCGCCTCT
CATGGCTTCATCGTCATCGCCCCTCAGTTATACTCTGTGGCCGGACCAGATACAAGCGAAGAAATCAAA
GCAACAGCCGCCATAATAAACTGGCTGCCGGAAGGGCTCCGGCAACTCCTTCCACCGCACGTGAACCCA
AACCTAATCAAAATAGCCCTCGCTGGCCACAGCCGCGGAGGCAAAACCTCCTTCGCTTTAGCCCAACAA
AAATCCCCAAAATTGTCAGCCCTAATCGGCCTGGATCCCGTCGACGGAACAGGGTCCGGCAAGCAAACC
CATCCCCCAGTCCTGAAATACATCCCCCAATCCTTGGATCTAGGGCTCCCTGTTCTGGTAATCGGGTCG
GGTCTGGGCGAATTGAAAAGAAATCCCCTGTTTCCCCCTTGCGCTCCAAAGGGAATCAACCACGAAGAG
TTTTTCAAGGAGTGCCGGAATCCGGCGTACTATTTTGTGGTGAAGGATTACGGGCATTTGGATTTGTTG
GATGATGAAACTGGAGGGATTAGAGGGAAGGCGAGCTACTGTTTGTGTAAAAATGGGGAATCTAGAGAG
CCGATGAGGAGGTTTGTTGGTGGGGCTGTGGTTGCGTTTTTGAAGGCTTATTTTGATGGGGAAGAAGGA
GATCTGAGAGCCATTGAAGATGGGGATTTGAGTTTGCCTATCCAACTTCAAACTGTTGAATCTCTTCTC
TGA

```
BgClh1   ......................................................................     0
BgClh2   ATGGGGGATGAAAAATTGAAATTGAAACTCCAACAAACCTCACTCGCCACCGCCACCGCCATGGCTATGG    70
Consensus

BgClh1   .................ATGGCGGCAGTAATAGTGGTGAAAGAAGAGGGGAAGCCAGTTTCAGCGGCGGT    53
BgClh2   CTACTGCTTCTTTTCCTCCGCCGTCGGCCACCGCCGGCTCTGCAATTTCGTCAAATGTCTTCGAGATCGG   140
Consensus                   g cg c g  a  g  g     g a     g      gt t  g g   g

BgClh1   GGATCCTAGTGAAGTTTTTGAAACAGGGAAATTTGAAGTGAGTACCATCACTGTGAAAACAGGCATATTT   123
BgClh2   AAAATTCAGCGCGGTGCTTGAAAAGTTCGAACCGGCTGGATGTTGCTCATCTGGCCGATTTCTTCCGGCT   210
Consensus  a    ag g  gt  ttgaaa      aa   g  g   gt  c    ctg    a           t

BgClh1   TCAGCTTCAAAGCCATTGCTTATCTTCACGCCGACCAACCCTGGTTCTTATCCAGTCATCTTGTTCCTTC   193
BgClh2   CCCCCGCCGAAGCCGCTGCTGATCGGCCGGCCTTCCGAAGCCGGAGAGTTCCCGGTGCTGCTGCTCATCC   280
Consensus c  c  c aagcc  tgct atc  c  gcc  cc a  c gg    t  cc gt  t  tg tc t c

BgClh1   ATGGCTTCTCCTGCGCTGGATCCTTCTACTCTGATTTCCTCAACCTTATAGCTTCCCATGGCTACATTAT   263
BgClh2   ATGGCTATCTCCTTTACAACACTTTCTACTCTCAGCTCATCCATCACATCGCCTCTCATGGCTTCATCTT   350
Consensusatggct    c          c ttctactct a  tc tc a c  at gc tc catggct cat  t

BgClh1   TGCTGCCCCACAGTTGTACGTGATGCCAACCACAAGTGAAATGAACGAAATAAACTCAGCATCAGAAGTA   333
BgClh2   G..TACTCTGTGGCCGGACCAGAT.......ACAAGCGAA......GAAATCAAAGCAACAGCCGCCATA   405
Consensus   t c c    g  g ac  gat       acaag gaa      gaaat aa  ca ca c g   ta

BgClh1   ATAACATGGTTGGCGTCGGGACTTGACCCATTACTCCCAGACAAAGTGAAAGGAGATATTTCAAAGCTAA   403
BgClh2   ATAAACTGGCTGCCGAAAGGCCTCCGGCACCTCCTTCCACCGCACGTGAACCCAAACCTAACCAAAATAG   475
Consensusataa  tgg tg cg   gg ct    c   t ct cca    a gtgaa   a a  t  c aa  ta

BgClh1   GTCTAGTGGGGCACAGCCGAGGTGGCAAAACAGCCTTTTCCTTAGCCTTAGGCTACGGCCGCCCTTCCCT   473
BgClh2   CCCTAGCCGGCCACAGCCGCGGCGGCAAAACTTCCTTCGCCTTAGCCCAACAAAAA.........TCCCC   536
Consensus  ctag  gg cacagccg gg ggcaaaac  cctt  ccttagcc  a    a          tccc

BgClh1   TCCCTTCTCCGCCGTAATCGGCGTCGACCCAGTAGCCGGAACCAAATACTTCCAGCCGG...AGCCCCAC   540
BgClh2   AAAATTGTCAGCCGTAATCGGGCTCGATCCCGTCGACGGAACAGGGTCCGGCAAGCAAACCCATCCCCCA   606
Consensus    tt tc gccgtaatcgg  tcga cc gt g cggaac    t c  c agc      a cccc

BgClh1   ATCCTAACCCCACTCTCCGAACCCTTCAACATTTCCGTACCCATCACAGTCATTGGAACCGGACTGGGCC   610
BgClh2   GTTCTGAAATACATCCCCCACTCGTTGGATCTAGGGCTCCCGGTTCTGGTAATCGGGTCGAGTCTGGGAG   676
Consensus t ct a      tc cc a  c tt  a  t     t cc  t    gt at gg  c  g ctggg

BgClh1   CCAAGAGAGCGAACCCAGTCACATGCCCTTGCGCCCCTGTTGGCTACAACCACGTTGAGTTTTTCCAAAA   680
BgClh2   AATTGAAAAGAAATCCACTGTTTCCCCCTTGCGCCCCAAAAGGAATCAACCACGAGGAGTTCTTTAAGGA   746
Consensus    ga a   aa cca t      cccttgcgcccc    gg   caaccacg  gagtt tt  a  a

BgClh1   AAGCAAGCCCAGTTGCGCCCACTTTGTAGCGGTGAATTACGGCCACATGGACATTCTAAACGACAACCCG   750
BgClh2   GTGCCGGAATCCGGCGTACTATTTTGTTGTGAAGGATTACGGGCATTTGGATTTGTTGGATGATGAAACT   816
Consensus  gc  g           c a tttgt g g  g attacgg ca  tgga  t  t  a ga  a  c

BgClh1   CCTGGGATGACAGGCTTTTTCACGAACATTGCTTGTAAGAATGGGAAAGGCCCAAGAAAGCTTATGAGGA   820
BgClh2   GGAGGGATTAGAGGGAAGGTGAGTTACTGTTTGTGTAAAAATGGGGAA...TCTAGAGAGCCGATGAGGA   883
Consensus   gggat a agg     t a   ac  t   tgtaa aatggg aa    c aga agc  atgagga

BgClh1   AGTGCTGCAGTGGGCTTGTGGTTGCTTCTTTGAAGGCTTATCTTGACAATGATGAATCATTTCTGAATGC   890
BgClh2   GGTTTGTTGGTGGGGCTGTGGTTGCGTTTTTGAAGGCTTATTTTGATGGGGAAGAAGGAGATTTGAGAGC   953
Consensus gt      gtggg  tgtggttgc t tttgaaggcttat ttga    ga gaa  a  t tga  gc

BgClh1   TATTTATATTGACCCTTCTATTGCTCCTGTTGAGCTAAATCCTGCTGAGGTTATTTACAAAACATCCTCC   960
BgClh2   CATTGAAGATGGGGATTTGAGCTTGCCTGTTCAACTTCAAACTGTTGAATCTTTTCTCTGA.........  1014
Consensus att a   tg    tt  a     cctgtt a ct  a  ctg tga   t tt  c  a

BgClh1   TCTAAATAA                                                                969
BgClh2   .........                                                               1014
Consensus
```

```
WmClh1   ............................................................ATGGCGGCAA     10
WmClh2   ATGGCTATGGCTACTGCTTCTTTTCCTCCGCCGTCGGCCACCGCGCGCTCTGCAGTTTCGTCCAATGTCT     70
Consensus                                                                  g

WmClh1   TAGTGGTGAAAGAAGCTAAGTCAGTTTTCGAAACAGGAAAATTTGAAGTAACTGCCATCACTGTAAAATC     80
WmClh2   TCGAGATCGGAAAATTCAACGCGGTGCTTGAAAAGGTCGAATCGGCTGGATGTTGCTCATCTGGCCGATT    140
Consensust g g t   a aa   aa  c gt  t gaaa  g   aat  g  g a  t  c    ctg    at

WmClh1   AGACATATTTTCAAGTTCAAAGCCATTGCTTATCTTCACACCGACCAACCCAGGTTCGTATCCCCTCATC    150
WmClh2   TCTTCCGGCTCCTCCGCCGAAGCCGCTGCTGATCGGCCGGCCTTCCGATGCCGGAGAGTTCCCGGTGCTG    210
Consensus         t c     c aagcc  tgct atc  c   cc  cc a  c gg   gt  cc  t  t

WmClh1   TTGTTCCTTCATGGCTTCTCCTGCGCCGGATCCTTCTACTCCCATCTCCTTCGCCCCATCGCTTCCCATG    220
WmClh2   CTGCTCATCCACGGCTATCTCCTTTACAACACTTTCTACTCTCAGCTTATCCATCACATCGCCTCTCATG    280
Consensus tg tc t ca ggct    c     c    c ttctactc ca ct  t c  c catcgc tc catg

WmClh1   GCTACGTTATTGCTGCCCCACAGTTGTACATGATGCCAACCACAAGTGAAATGGAAGAAATAAAGTCAGC    290
WmClh2   GCTTCATCGTCATCGCCCCTCAGTTATACTCTGTGGCCGGACCAGATACAAGCGAAGAAATCAAAGCAAC    350
Consensusgct c t  t    gcccc cagtt tac    tg c     ca  t  aa  gaagaaat aa  ca c

WmClh1   ATCAGAAGTGATAACATGGTTGCGGTCGGGACTTAACCCATTACTCCCAAACGATGTAAAAGGAGATATT    360
WmClh2   AGCCGCCATAATAAACTGGCTGCCGGAAGGGCTCCGGCAACTCCTTCCACCGCACGTGAACCCAAACCTA    420
Consensusa c g   t ataa  tgg tgc g   gg ct    c a t ct cca    a gt aa   a a  t

WmClh1   TCAAAGCTAAGTCTAGTGGGCCATAGCCGAGGTGGCAAAACAGCCTTTTCCTTAGCCTTAGGCTACGGCC    430
WmClh2   ATCAAAATAGCCCTCGCTGGCCACAGCCGCGGAGGCAAAACCTCCTTCGCTTTAGCCCAA.........C    481
Consensus   aa  ta   ct g  ggcca agccg gg ggcaaaac  cctt  c ttagcc  a         c

WmClh1   GCCCTTCCCTTCCCTTCTCCGCGGTCATCGGCATCGACCCAGTCGCCGGAACCAAGTGTTTCCAGC...C    497
WmClh2   AAAAATCCCCAAAATTGTCAGCCCTAATCGGCCTGGATCCCGTCGACGGAACAGGGTCCGGCAAGCAAAC    551
Consensus     tccc     tt tc gc  t atcggc t ga cc gtcg cggaac   gt    c agc   c

WmClh1   CCACCCCCACATCCTAACCCCACTCTCCGAACCCTTCAACATTTCCGCACCCATCACAGTCATTGGAACC    567
WmClh2   CCATCCCCCAGTCCTGAAATACATCCCCCAATCCTTGGATCTAGGGCTCCCTGTTCTGGTAATCGGGTCG    621
Consensuscca cccc   tcct a      tc cc aa cctt  a  t       cc  t    gt at gg  c

WmClh1   GGGCTGGGTCCCGAGAAGGCGAACCTAGTCACATGCCCTTGCGCCCCCGATGGCTACAACCACGCTGCGT    637
WmClh2   GGTCTGGGCGAATTGAAAAGAAATCCCCTGTTTCCCCCTTGCGCTCCAAAGGGAATCAACCACGAAGAGT    691
Consensusgg ctggg      gaa    aa c   t      cccttgcgc cc  a gg   caaccacg  g gt

WmClh1   TTTTCCAAAAGAGCAAGCCCACTCGCGCTCACTTTGTGGCGGTGGACTACGGGCACATGGACGTTCTGAA    707
WmClh2   TTTTCAAGGAGTGCCGGAATCCGGCGTACTATTTTGTGGTGAAGGATTACGGGCATTTGGATTTGTTGGA    761
Consensusttttc a  ag gc  g    c        a tttgtgg g  gga tacgggca  tgga  t  tg a

WmClh1   CGACAACCCGCCTGGGATTACGGGCCTTTTCACCAACATTGCTTGTAAGAACGGGAAGGGTCCAAGAGAG    777
WmClh2   TGATGAAACTGGAGGGATTAGAGGGAAGGCGAGCTACTGTTTGTGTAAAAATGGGGAA...TCTAGAGAG    828
Consensus ga  a  c    gggatta  gg       a c ac  t   tgtaa aa ggg a     c agagag

WmClh1   CTTATGAGGAAGTGCTGCAGTGGACTCGTGGTTGCTTCCTTGAAGGCTTATCTTGACAATGATCAATCAA    847
WmClh2   CCGATGAGGAGGTTTGTTGGTGGGGCTGTGGTTGCGTTTTTGAAGGCTTATTTTGATGGGGAAGAAGGAG    898
Consensusc  atgagga gt      gtgg    gtggttgc t  ttgaaggcttat ttga    ga  aa  a

WmClh1   TTCTGAATGCCATTTATGTTGACCCTTCCATTGCTCCCGTGGACCTAAATCCTGTGGAGCTTATTTACAA    917
WmClh2   ATCTGAGAGCCATTGAAGATGGGGATTTGAGTTTGCCTATCCAACTTCAAACTGTTGAATCTCTTCTCTG    968
Consensus tctga  gccatt a g tg    tt  a t   cc  t  a ct  a  ctgt ga   t tt  c

WmClh1   ACCATCCACTGCACAGGGGCTGATCTATTAA                                           948
WmClh2   A..............................                                           969
Consensusa
```
